# The frequency of Yq microdeletion in azoospermic and oligospermic Iranian infertile men

**Published:** 2013-06

**Authors:** Mohammad Ali Zaimy, Seyyed Mehdi Kalantar, Mohammad Hasan Sheikhha, Tahere Jahaninejad, Hossein Pashaiefar, Jalal Ghasemzadeh, Mahnaz Zahraei

**Affiliations:** 1 *Research and Clinical Center for Infertility, Shahid Sadoughi University of Medical Sciences, Yazd, Iran.*; 2 *Department of Genetics, Faculty of Medical Sciences, Shahid Sadoughi University of Medical Sciences, Yazd, Iran.*

**Keywords:** *Male infertility*, *Multiplex polymerase chain reaction*, *AZF microdeletion*

## Abstract

**Background: **About 15% of couples have infertility problems which 40% of them are related to the male factors. Genetic factors are candidate for about 10% of male infertility conditions. Among these, AZFa, AZFb, AZFc and AZFd regions on the Yq are considered most important for spermatogenesis. Microdeletions of these regions are thought to be involved in some cases of azoospermic or oligospermic infertile men.

**Objective:** We studied the prevalence of AZF microdeletions among Iranian infertile men with non-obstructive azoospermia and oligospermia.

**Materials and Methods: **A total of 50 Iranian azoospermic and oligospermic infertile men were selected for case group and 50 men with normal spermogram as control group. The molecular study of Y chromosome microdeletions was done by multiplex polymerase chain reaction (M-PCR) method by using of 13 sequence tagged site (STS) markers from AZF region.

**Results:** Four (8%) patients showed Y chromosome microdeletions among case group, deletion in AZFc region was the most frequent (80%) followed by AZFb (20%), in AZFa and AZFd region we did not detect any deletions. No deletion was detected in control group; the ratio of Y chromosome microdeletion in azoospermic men was higher than this ratio in oligospermic men [19% (3/16) among azoospermic men and 3% (1/34) among oligospermics]. Serum FSH level in men with microdeletions was higher than this level in men with no deletions (p=0.034).

**Conclusion:** Because of relatively high prevalence of microdeletions on the long arm of Y chromosome among Iranian azoospermic and oligospermic patients, screening of this microdeletion may be advised to infertile men particularly azoospermic and oligospermic men before using assisted reproductive treatments.

## Introduction

Infertility is the inability to conceive after one year of unprotected intercourse (1). Infertility affects approximately 10-15% of all married couples attempting pregnancy in the general population, and today it is a major public health problem. Male factor is responsible for 40% of the cases approximately and in 20% of cases both man and woman are affected (2, 3). Male infertility can be caused by many different factors, such as infection, varicose, endocrine disorders, spermatic duct obstruction, anti-sperm antibodies, systemic diseases, testicular cancer, testicular trauma, malnutrition, genetic defects and environmental conditions (2, 4, 5). It is estimated that 40-50% of these men have quantitative or qualitative abnormality in their sperm production. In more than 60% of these cases the origin of testicular dysfunction is unknown (6). It has been shown that the Y chromosome is involved in spermatogenesis. Since 1976 it was established that some deletions in long arm of Y chromosome is associated with sperm production failure (7).

In the last few years, using molecular methods, the loci involved in production and differentiation of sperms have been identified. Male-related genes such as sex-determining region of Y-chromosome (SRY) and several other spermatogenesis-related genes are accumulated in Y chromosome (8). The Y chromosome has been subdivided into 7 deletion intervals, each of these intervals consists of subintervals (A, B, C, etc.) (9) 

In 1992, Vollrath *et al* constructed a map of Y chromosome with 43 interval deletions that consist of a region of sequence tagged sites (STS) which span to the both arms of Y chromosome. Critical genes for spermatogenesis are located in deletion interval 6 and deletion interval 5 also on long arm of Y chromosome. 

Deletion in the azoospermia factor (AZF), region is associated with azoospermia and oligospermia. The AZF region has at least 3 loci-AZFa, AZFb, and AZFc which are required for normal spermatogenesis (3). In some text a fourth region has been suggested to be existing between AZFb and AZFc region, and is termed as AZFd (10).

DFFRY and DBY are two main genes in AZFa region. RBMY, PRY, and CDY2 are protein-encoding genes families on the AZFb region that are expressed in the testis only. BPY2, CDY, DAZ, CSPG4LY, GOLGAZLY are genes on AZFc region which are expressed only in testis. This region also has some genes that they have not testis specific expression (11, 12). 

Using assisted reproductive technologies (ART) such as in vitro fertilization (IVF) and intra cytoplasmic sperm injection (ICSI) for treatment of male infertility may result in the passing of Y chromosome microdeletions to the sons, resulting in the persistence of infertility problem over the next generations (13-15). 

In this study we evaluated the frequency of AZF deletions among Iranian azoospermic and oligospermic infertile men using multiplex polymerase chain reaction (M-PCR) method.

## Materials and methods

This research was a case control study, which was carried out in Yazd Research and Clinical Center for Infertility in 2012. In total 49 infertile men were randomly selected from couple attending to Yazd Research and Clinical Center for Infertility as case group. Inclusion criteria were azoospermic or oligospermic men with no anatomical or syndromic abnormalities. According to WHO criteria, men that their sperm count was less than 2x10^6^/ml were grouped as oligoosperms. For control group, 50 normozospermic men were selected according to semen analysis which was performed according to WHO normal standard parameters. 

Exclusion criteria were men with anatomical or syndromic abnormalities. Informed consent was taken from all patients. This study was confirmed by ethical board of Yazd Research and Clinical Center for Infertility. Financial support was provided by Yazd Research and Clinical Center for Infertility.

**Molecular** **analysis**

Genomic DNA was extracted from leukocytes of peripheral blood by salting out method (16). According to the European Academy of Andrology (EAA), the European Molecular Genetics Quality Network (EMQN), and previous studies that were done in Iran, we selected a series of 13 sequence tagged sites (STSs) markers (17, 18). The STS markers were sY182 for AZFa, sY133 for AZFb, sY255, sY254, sY146, sY158, sY238, sY155, sY277, sY272, sY283, sY157 for AZFc and sY153 for AZFd. Because of high incidence of Y chromosome microdeletion in AZFc region we selected 10 STS in this region to find the best and the most informative STSs groups for screening of this region. 

The sequence of primers is shown in table I. Seven set of multiplexes were designed as follows:

- Multiplex 1: sY238, sY153
- Multiplex 2: sY133, sY255, sY272
- Multiplex 3: sY283, sY146
- Multiplex 4: sY155, sY158
- Multiplex 5: sY254, sY182
- Multiplex 6: sY157, sY182
- Multiplex 7: sY277, sY182.

The mixture of materials in PCR master mix was: 

PCR 5X reaction buffer: 2.5µl (Faza pajouh Iran)

dNTPs mixture: 1.0µl (Faza pajouh Iran)

MgCl_2_:1.3µl (Faza pajouh Iran)

Primers 2-4 µl (Faza pajouh Iran)

H_2_O:14.8 µl 

Taq DNA polymerase: 0.7µl (Faza pajouh Iran)

Genomic DNA sample: 0.7µl. 

In this study we used two thermal programs, one as normal PCR:

1/ 95^o^C, 60s

2/ 95^o^C, 30s, 56^o^C, 30s, 72^o^C, 30s for 23 cycles

3/ 72^o^C, 60s

In some reactions we use touchdown thermal PCR program in that the first annealing temperature was 63^o^C and the final annealing temperature was 57^o^C in 17-19 cycles. The annealing temperature in different reaction according to primers Tm could be changed.

**Statistical analysis**

X^2^ test was done to compare differences between the case and control group. The statistical analysis was performed with SPSS 16 statistical software. The p-value under 0.05 was considered as significant difference.

## Results

As described in methods, a total of 50 infertile men were selected in this study, 16 (16/50=32%) of whom were azoospermic males and 34 (34/50=68%) were oligospermic men. 

The total percentage of microdeletions detected in case group was 8% (4/50). AZFc was the most deleted region (4/5=80% of deletions) that followed by AZFb (1/5=20% of deletions), AZFa and AZFb have no deletions. No men in control group show microdeletion in Yq chromosome. The percentage of Y chromosome microdeletion in azoospermic men was higher than this percentage in oligospermics [19% (3/16) among azoospermic men and 3% (1/34) among oligospermics]. Serum FSH level in men with microdeletions was higher than men that have no deletions (p=0.034). 

There was not a significant difference between men with Y chromosome microdeletions and men no deletions age (p=0.60).

**Table I:** STSs name and their primer sequences

**STS**		**Primers**
SY254	NCBI UniSTS code: 54754	5- GGGTGTTACCAGAAGGCAAA -3
		5-GAACCGTATCTACCAAAGCAGC-3
SY255	NCBI UniSTS code: 166986	5-GTTACAGGATTCGGCGTGAT-3
		5-CTCGTCATGTGCAGCCAC-3
SY153	NCBI UniSTS code: 47914	5-GCATCCTCATTTTATGTCCA-3
		5-CAACCCAAAAGCACTGAGTA-3
SY133	NCBI UniSTS code: 21574	5-ATTTCTCTGCCCTTCACCAG-3
		5-TGATGATTGCCTAAAGGGAA-3
SY155	NCBI UniSTS code: 21812	5-ATTTTGCCTTGCATTGCTAG-3
		5-TTTTTAAGCCTGTGACCTGG-3
SY146	NCBI UniSTS code: 6871	5-ACAAAAATGTGGCTCAGGGA-3
		5-AAATAGTGTGCCCACCCAAA-3
SY182	NCBI UniSTS code:64826	5-TCAGAAGTGAAACCCTGTATG-3
		5-GCATGTGACTCAAAGTATAAGC-3
SY157	NCBI UniSTS code: 39767	5-CTTAGGAAAAAGTGAAGCCG-3
		5-CCTGCTGTCAGCAAGATACA-3
SY158	NCBI UniSTS code: 36582	5-CTCAGAAGTCCTCCTAATAGTTCC-3
		5-ACAGTGGTTTGTAGCGGGTA-3
SY283	NCBI UniSTS code: 93132	5-CAGTGATACACTCGGACTTGTGTA-3
		5-GTTATTTGAAAAGCTACACGGG-3
SY238	NCBI UniSTS code: 2449	5-AACAAGTGAGTTCCACAGGG-3
		5-GCAAAGCAGCATTCAAAACA-3
SY277	NCBI UniSTS code: 93131	5-GGGTTTTGCCTGCATACGTAATTA-3
		5-CCTAAAAGCAATTCTAAACCTCCAG-3
SY272	NCBI UniSTS code: 166987	5-GGTGAGTCAAATTAGTCAATGTCC-3
		5-CCTTACCACAGGACAGAGGG-3

**Table II:** Some features of 4 patients with Y chromosome microdeletions

**Patient No.**	**Age (Year)**	**Sperm count x10** ^6^ **/ml**	**STS**	**AZF regions**	**Infertility duration (month)**	
9	28	0	sY238	AZFc	18
27	44	0	sY255, sY254	AZFc	168
30	24	0	sY133	AZFb	46
33	34	4	sY254	AZFc	12

**Figure 1:**
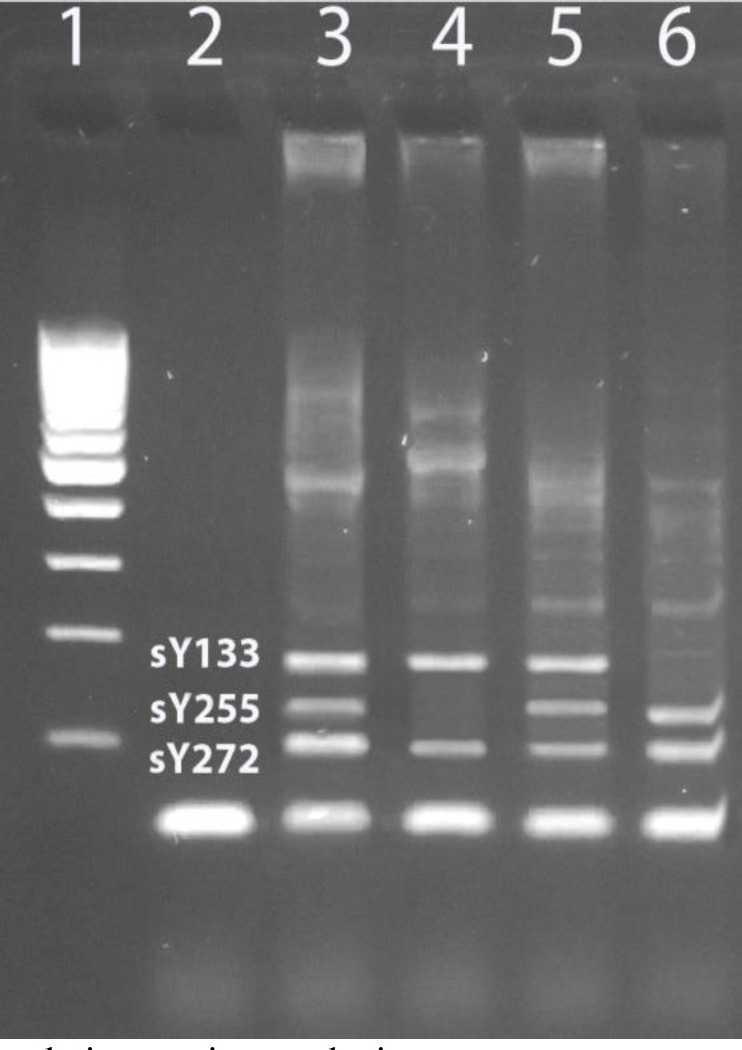
Results from multiplex polymerase chain reaction analysis.

**lane 1 shows 100bp DNA marker.**

**lane 2 shows normal woman.**

**lane 3 shows a normal man.**

**lane 4 shows patient with deletion in sY255.**

**lane 5 shows patient with no deletion.**

**lane 6 shows patient with deletion in sY133.**

## Discussion

Deletions of AZF regions are deletions of the euchromatin part of the Yq chromosome. It is thought that these deletions of Y chromosome can damage genes in this region that are responsible for the proper course of spermatogenesis. Many factors such as somatic and sex chromosome genes interactions are candidate to have role in the normal spermatogenesis. The AZF deletions are the most frequent cause of spermatogenetic failure (8). After Klinefelter’s syndrome, Yq microdeletions are the second most frequent and important spermatogenesis genetic disorder in male infertility. Previous studies have reported that Yq microdeletions changed from 1-55% between infertile men, but most studies have revealed this ratio below 15% (19). 

In our study the frequency of microdeletion in azoospermic and oligospermic men was 19% among azoospermic men and 3% among oligospermic men that is similar to the range of the published data (20). Some studies that were done in Iran have reported different results from our study. Omrani *et al* showed that the incidence of microdeletions among azoospermic patients in North West of Iran was 24.2%; Malekasgar *et al *indicated that the incidence of Yq microdeletions in South West of Iran was higher than international frequency. They found microdeletions in 52% (16, 21).

These variations in frequencies could be due to different origins of the studied population and differences in the study design including the composition of the study population and different STSs selection. Mirfakhraie *et al *showed that the incidence of microdeletion in Yq chromosome is 12% that this result is similar to ours (17). In table III we compared our results with other similar studies. With use of mentioned STSs we reported the most frequent microdeletions in AZFc, followed by AZFb and no deletion in AZFa and AZFd that is similar to many past studies such as Mirfakhraie *et al* who reported the most frequent microdeletions in AZFb region followed by AZFc.

Controversy in Y chromosomal microdeletions of infertile men arises from this fact that Yq microdeletions will be transmitted by ICSI and cause the infertility problem in sons. This possibility has been described previously (22). Screening of Yq microdeletions can help medical stuff to give informative counseling to couples with these deletions that want to use Because of this probability ethical consequences should be one of the important dimensions of these techniques. 

**Table III:** Percentage of Yq microdeletion in different study

**Study**	**Year**	**Percentage of microdeletios**
Balkan *et al.*	2008	1.3%
Vogt *et al.*	1996	3%
Fernanda *et al.*	2011	4.2%
Qureshi *et al.*	1996	8%
DADA *et al.*	2003	9.63%
Mirfakhraie *et al.*	2010	12%
Reijo *et al.*	1995	13%
Kobayashi *et al.*	1994	19%
omrani *et al.*	2006	24.2%
Al-faisal *et al.*	2010	27.9%
Malekasgar *et al.*	2008	52%
This study	2012	8%

## Conflict of interest

The authors declare that there is no conflidt of interest.
